# One byte at a time: evidencing the quality of clinical service next-generation sequencing for germline and somatic variants

**DOI:** 10.1038/s41431-019-0515-1

**Published:** 2019-09-30

**Authors:** Maria Weronika Gutowska-Ding, Zandra C. Deans, Christophe Roos, Jukka Matilainen, Farrah Khawaja, Kim Brügger, Jo Wook Ahn, Christopher Boustred, Simon J. Patton

**Affiliations:** 1 European Molecular Genetics Quality Network (EMQN), Manchester Centre for Genomic Medicine, St Mary’s Hospital, Manchester, UK; 2 Genomics Quality Assessment (GenQA), Department of Laboratory Medicine, Royal Infirmary of Edinburgh, Little France Crescent, Edinburgh, UK; 3 Euformatics Oy, Tekniikantie 12, Espoo, Finland; 4 Department of Informatics, University of Bergen, Bergen, Norway; 5 Genetics Laboratories, Guy’s and St Thomas’ NHS Foundation Trust, London, UK; 6 Genomics England, London, UK

**Keywords:** Genetic testing, Genome informatics, Cancer genomics, Cancer genomics, Next-generation sequencing

## Abstract

Next-generation sequencing (NGS) is replacing other molecular techniques to become the de facto gene diagnostics approach, transforming the speed of diagnosis for patients and expanding opportunities for precision medicine. Consequently, for accredited laboratories as well as those seeking accreditation, both objective measures of quality and external review of laboratory processes are required. External quality assessment (EQA), or Proficiency Testing (PT), can assess a laboratory’s service through an independent external agency, the EQA provider. The analysis of a growing number of genes and whole exome and genomes is now routine; therefore, an EQA must be delivered to enable all testing laboratories to participate. In this paper, we describe the development of a unique platform and gene target independent EQA scheme for NGS, designed to scale from current to future requirements of clinical diagnostic laboratories testing for germline and somatic variants. The EQA results from three annual rounds indicate that clinical diagnostic laboratories are providing an increasingly high-quality NGS service and variant calling abilities are improving. From an EQA provider perspective, challenges remain regarding delivery and performance criteria, as well as in analysing similar NGS approaches between cohorts with meaningful metrics, sample sourcing and data formats.

## Introduction

The decrease in cost and wide availability of next-generation sequencing (NGS) has enabled the application of this technique in numerous aspects of routine clinical medical diagnostics and NGS is therefore replacing other molecular techniques to become the de facto standard, transforming the speed of diagnosis for patients and expanding opportunities for precision medicine [1–3].

Whatever NGS approach is deployed, clinical diagnostic laboratories must provide the correct test result, accurately interpreted, to the right patient within an appropriate time-frame. Any errors in this process may result in inappropriate clinical decisions for patient management, treatment, and in the case of inherited disease, could have an impact beyond the patient’s own diagnosis.

NGS also presents challenges when implemented into routine clinical diagnostic practice not only in terms of cost implications of validation, but also knowledge and capacity for data analysis. Consequently, more efficient and structured sharing of validation and quality control data, as well as variant annotation and analytical strategies could help minimise wasteful duplication and speed progress in identifying new genomics-based tests, helping to translate them into the clinic [4]. This creates the need for multiple skill sets and reconfiguration of current diagnostic processes. Best practice guidelines (BPG) for NGS-based tests set comprehensive requirements on a laboratory’s own Internal Quality Control (IQC) practices not only for the sequencing but also quite extensively for the complex bioinformatics workflows [5–8]. For accredited laboratories, and those seeking accreditation, objective measures of quality such as external review of laboratory processes are critical.

Medical laboratory accreditation to a recognised standard, for example ISO 15189 [9], is one such system of external review. ISO 15189 accreditation requires that laboratories regularly participate in EQA for all tests performed. The objectives of EQA are to assess the standard of the tests provided by a laboratory through interlaboratory compatibility, promote high-quality testing through education and continued improvement, and to recognise the competence of medical laboratories by their service users, regulatory authorities, and accreditation bodies.

The widespread introduction of NGS across different testing strategies has resulted in clinical laboratories analysing a growing number of targets across a broad range of conditions. Therefore, since an EQA scheme for NGS cannot cover every gene variant, it must be delivered as a technical assessment, and needs to be platform and gene target agnostic. Critically, it must be able to accommodate future changes in technology and/or methodology.

A successful NGS EQA creates or has access to a thoroughly validated reference truth set against which participating laboratories can be measured. This requirement brings challenges in providing large quantities of EQA material where the validated EQA truth set is unknown to the participating laboratories. In this paper, we describe the development of a unique platform and gene target independent EQA scheme for NGS, designed to meet current and future requirements of clinical diagnostic laboratories testing for germline and somatic variants. We also describe a novel process for creating an EQA participant consensus truth set.

## Materials and methods

### Laboratory participant selection

The EQA scheme was provided annually from 2015 to 2017 as a collaboration between two established providers: the European Molecular Genetics Quality Network (EMQN) and the Genomics Quality Assessment (GenQA, previously known as United Kingdom National External Quality Assessment Service (UK NEQAS) for Molecular Genetics). Participation was voluntary and unrestricted; laboratories enrolled via the EMQN [10] or GenQA [11] websites.

### Scheme design

**Fig. 1:**
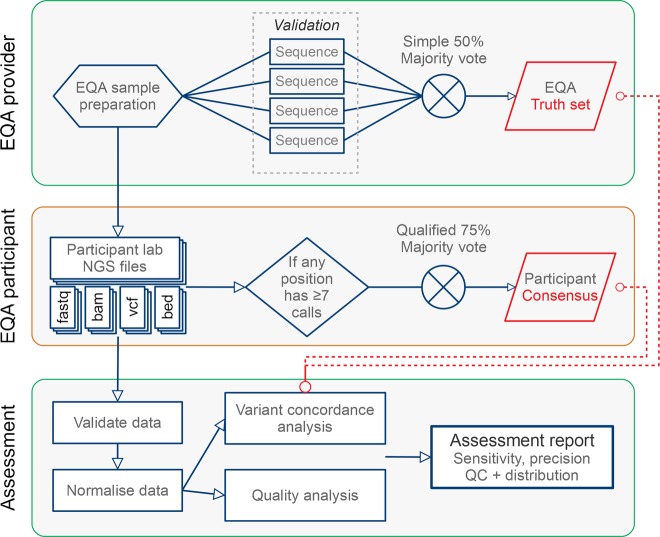
Schematic illustration of the NGS EQA workflow

The EQA scheme was designed to be agnostic to the testing context, platform, and gene target(s); two versions were provided, germline and somatic as distinct assessments, each one with its own sample. Laboratories were required to test the EQA sample and generate data following their standard operating procedures. It was not specified how, or which genes should be tested; single genes, gene panels, exomes and whole genomes were acceptable. Following feedback from the 2015 round, enhancements were made to the online submission platform so that participants could submit up to three separate datasets each for the 2016 and 2017 germline and somatic versions, enabling them to focus on different panels and/or whole genome sequencing (WGS), or using different platform/techniques (Fig. 1).

### Sample materials

The EQA materials differed depending on the scheme version. For the germline scheme, each laboratory received 10 µg of genomic DNA (g.DNA) from a lymphoblastoid cell line (Coriell Cell Repositories, Camden, New Jersey, USA; 2015—cell line NA00536; 2016—cell line NA06926; 2017—cell line NA21070) extracted using the salting out procedure [12]. For the somatic scheme, each laboratory received identical reference materials derived from a genetically engineered cell line containing somatic variants, manufactured by Horizon Discovery Plc, Cambridge, UK [13], or patient-derived materials. In 2015, 5 µg of DNA extracted using the Promega Maxwell 16 FFPE plus LEV DNA Purification Kit (Promega cat# AS1135) from a formalin fixed, paraffin embedded (FFPE) cell line product (HD200 containing a multiplex of variants in three different cell line backgrounds—SW48, RKO and HCT116) was supplied. In 2016, a 10 µm FFPE section containing a cell line with somatic variants (Horizon Discovery Plc. cell line HD301—KRAS Gene-Specific Multiplex Reference Standard) was supplied, along with a 10 µm FFPE section from the matched normal control i.e. germline sequence (Horizon Discovery Plc. cell line HD320—PI3KCA Wild Type Reference Standard). In 2017, 150 ng of DNA extracted using the Qiagen QiaAmp DNA FFPE kit from FFPE sections from a breast tumour was supplied along with 150 ng of DNA extracted using the same method from matched normal lymph node from the same patient.

### Defining the germline variant EQA truth set

Prior to distribution, the 2015 and 2016 germline samples were independently validated by four different laboratories; one using WGS (1× Illumina Hi-Seq), and the remaining three using Whole Exome Sequencing (WES) (2× Illumina Hi-Seq, 1× ThermoFisher/Life Technologies Proton). A variant was included in the EQA truth set if called by at least two validating laboratories due to differences in coverage between captures. In 2017 no predefined truth set was generated as the only truth set applied was the one obtained through a process derived from the consensus of the submitted variants (see below: defining the participant consensus truth sets).

### Defining the somatic variant EQA truth set

The 2015 and 2016 somatic EQA samples were independently validated by the manufacturer using WES on an Illumina Hi-Seq platform along with the Droplet Digital PCR™ (ddPCR) confirmation and quantification of the allelic frequency of the engineered variants. For the somatic scheme, the list of ddPCR-confirmed variants reported by the vendor of the reference sample (Horizon Discovery Plc.) was used as the EQA truth set.

### Assessable regions of the EQA truth sets

The assessable regions determined the genomic regions in which variants were assessed. Variants near 5′ and 3′ splice sites often affect splicing, but the effects of these variants on splicing and disease have not been fully characterised beyond the two ‘essential’ splice nucleotides flanking each exon. For the germline scheme, these regions were restricted to the protein-coding exons extended by two base pairs (bp) (UCSC Table Browser, GRCh37/hg19, https://genome.ucsc.edu/cgi-bin/hgTables?command = start) into the intronic regions. Since then further data are available and there are good reasons to include at least 8 bp in the future [14]. For the somatic scheme, the assessable regions comprised the locations of the known variants in the somatic variants EQA truth set without extension. In other words, the truth set was only composed of the collection of features exactly covering the somatic EQA truth set variants.

### Defining the participant consensus truth sets (germline and somatic)

In 2016 and 2017 a participant consensus truth set was derived by using the data provided by the participants’ submitted unfiltered (PASS) variants as extracted from the Variant Calling Files (VCF) [15]. These consensus sets were established by combining all of the submitted variants that simultaneously satisfied two conditions: (a) the position of the variant was in the region of interest (ROI) of at least seven participants, and (b) at least 75% of these participants agreed on the variant call at the position. When creating the participant consensus, the submissions were normalised by splitting multi nucleotide polymorphisms (MNPs) into single nucelotide polymorphisms (SNPs) and left-aligning indels. In 2016 and 2017 the participant consensus truth set was primarily used in assessing the participants’ submitted variants. In 2016 the pre-established EQA truth sets were used as fall-back as described in the section on concordance analysis. In 2017, only the consensus truth set was available for participant assessment. The 2017 participant consensus truth set for the germline scheme submissions using the GRCh37 reference genome was checked by evaluating the public WGS data set for the NA21070 subject available from the Personal Genomes Project (https://my.pgp-hms.org/profile/huC30901) against it. The WGS (from 2011) reached a sensitivity 99.0% and precision of 94.9% for a total of 21,161 true positives (TP), 1127 false positives (FP) and 224 false negatives (FN). A random selection of variants present in the consensus but not the PGP data, and vice versa (10 of each) were manually curated by a registered clinical scientist examining aligned reads using IGV. This showed that the majority of variant calls unique to the consensus were likely to represent true variants missing from the PGP data. Variant calls unique to the PGP data were more complex as some of these calls appeared to have been missed by participants and some by PGP. A more in-depth investigation is beyond the scope of this paper but will be addressed in a follow-up publication.

### Assessable regions of the consensus truth sets

In 2016 the assessable region was the same for the consensus truth set as for the EQA truth set for both germline and somatic. In 2017 the assessable region of the germline scheme was set to those protein-coding exons in which at least one consensus variant was included, extended by 2 bp. For the 2017 somatic scheme, any region further than 1000 bp away from the nearest reported variant was excluded from the participants’ ROI. This was done to correct for the large proportion of submissions where the participants had reported much larger ROI than where they had actually performed variant calling.

### Participant result submissions

Laboratories were required to submit results online to the EQA providers as a VCF abiding to the VCF version 4.1 or 4.2 standard [15] for their ROI declared in a BED file. The validity of the VCF and BED file format was automatically confirmed upon online submission, enabling immediate feedback to the submitter and amendment of the file by the participant, if required. In addition, two optional data types were requested for each submission: the raw data as compressed FASTQ and the alignment as BAM file. In the 2015 scheme, laboratories were also asked to submit a table of all the genes tested and the transcripts used. This requirement was amended in the 2016 scheme and laboratories were asked to submit a BED file for their ROI instead. From the 2016 and 2017 schemes it was also possible to further limit the ROI from the BED file down to named genes, targets, hot spots, and/or variants through either an inclusion or an exclusion operation on the BED file. Participants were required to complete an online form describing the sequencing approach(es) employed, the bioinformatics pipeline(s), and the internally defined quality thresholds applied.

In order to simplify and triage the data submission process, as well as to improve storage and quality control (QC) aspects of the EQAs, the data collection, storage, and analysis was contracted out to a commercial bioinformatics company, Euformatics, (Espoo, Finland) [16].

### Concordance analysis

Variant comparison was performed on SNPs and small indels (<50 bp) submitted by the participant in the intersection of the assessable regions (described above) and the submitted ROI. Before comparison, MNPs were split into SNPs and indels were normalised by left alignment using ‘bcftools norm’ to ensure that the comparison was correct to their minimal representation. In 2016, variants were compared against the EQA truth set augmented with the participant consensus truth set. Variants were primarily compared against the EQA truth set, but if there was a difference between the EQA truth set and the participant consensus where there was a strong consensus (at least seven7 participants targeting the position, with at least 75% of the variant calls in agreement with each other) the participant consensus was used as the truth. In 2017 only the consensus truth set was used for all concordance analysis. When comparing variants in the germline scheme, it was required that the called genotype matches. When comparing variants in the somatic scheme, it was required that the called alternate allele matches. Each comparison result was assigned to one of four categories:

- Agree (TP): The submitted variant was identical to the expected variant in the EQA truth set, or participant consensus truth set.
- Disagree (FP, FN): The submitted variant was different to the expected variant in the EQA participant consensus set, or the EQA truth set.
- Extra (FP): The submitted variant was in a location where none was expected, neither by the EQA participant consensus set, or the EQA truth set
- Missing (FN): No submitted variant was found in a location where one was expected according to the EQA participant consensus set, or the EQA truth set.

### Quality metrics analysis

Participant data for each sample were also analysed in the 2016 and 2017 scheme for a number of different quality metrics selected based on the EuroGentest and College of American Pathology (CAP) best practice guidelines [5, 6]. The specific metrics assessed from each file type were: (a) FASTQ: Average base quality (Phred quality score), bases above Q20 and Q30; (b) BAM: Reads uniformity (%), off target (%), error rate on target, coverage at 20× (%) and at 30× and (in 2017 somatic) 500× (%), insert size average and standard deviation (for paired-end data only); (c) VCF: Ti/Tv ratio, quality of calls, SNP and indel counts, Het:Hom ratio for both SNPs and indels.

## Results

### Laboratory participation

**Fig. 2:**
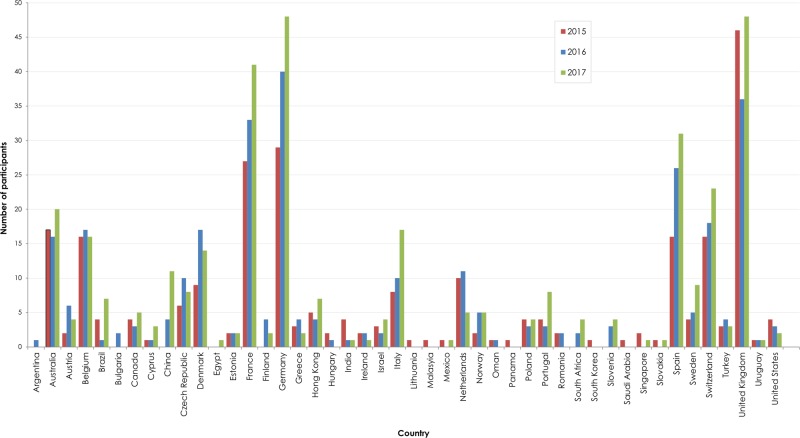
Country of origin of laboratories participating in the 2015, 2016 and 2017 EQA runs

Participant numbers increased from 182 in 2015, 213 in 2016 to 246 in 2017 for the germline scheme, and from 30 to 59 to 68 for the somatic scheme, respectively. In total, 423 different laboratories from 48 countries participated over the 3 years with the majority (75.6%) participating from 11 different countries (Fig. 2).

### Consensus sequences

In 2016, the germline scheme truth set contained 22,922 SNPs and 591 indels (maximum size 35 bp, median length 3 bp) and the somatic scheme truth set contained six SNPs and no indels. In 2017, the participant consensus for germline scheme submissions using the GRCh37 reference genome contained 24,344 SNPs and 712 indels (maximum size 45 bp, median size 3 bp), the participant consensus for germline scheme submissions using the GRCh38, introduced in 2017, reference genome contained 24,222 SNPs and 740 indels (maximum size 60 bp, median size 3 bp) and the participant consensus for somatic scheme submissions (GRCh37 only) contained 196 SNPs and one indel (deletion of size 3 bp).

### Laboratory practice

**Fig. 3:**
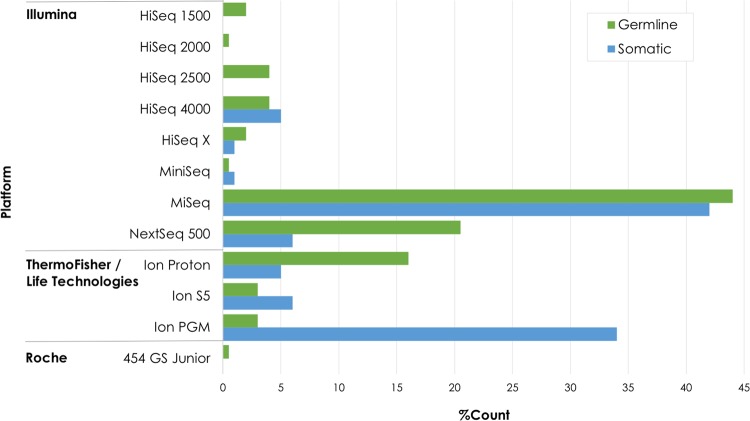
Different NGS platforms used by somatic and germline participants in all three EQA runs

Illumina NGS platforms were the most frequently used (72% in 2015, 73% in 2016, 80% in 2017) followed by ThermoFisher/Life Technologies Ion Torrent platforms (25% in 2015, 26% in 2016, 19% in 2017) while a small number of laboratories used the Roche 454 platform only in 2015 (1.45%). The ThermoFisher/Life Technologies Ion Torrent platforms were preferred by laboratories doing somatic variant testing (Fig. 3). The majority of the sequencing (64%) was bi-directional (paired-end) sequencing.

Most laboratories (69%) used a commercial kit for DNA capture or amplification. There was considerable variation in the kits used, of which 72% were commercially available and 28% custom designed for the purpose of the laboratory test (Supplemental data, Table 1).

There was wide variation in the minimum read depth required to report a variant. In both schemes, 69% of all participant laboratories confirmed that they would report a diagnostic test result without confirming it by a second, orthogonal method if it met their minimum read depth and other quality metric thresholds.

During the 2016 and 2017 EQA runs, over 19,000 unique genes were sequenced. Some laboratories changed their strategy between the EQA runs to performing WGS suggesting that this approach may be gaining favour over WES. Notwithstanding, in all runs the majority (89%) of laboratories tested small panels of up to 20 genes. *BRCA1* and *BRCA2* were the most frequently tested genes in the germline scheme, while other oncogenes such as *EGFR*, *KRAS* and *NRAS* were the most frequently tested in the somatic scheme.

Many different bioinformatics approaches including commercial software and in-house pipelines, for all or part of an analysis, were employed. The majority of participants (91%) used in-house bioinformatics pipelines (using self-made or commercial software tools), with 8% outsourcing bioinformatics processing to another laboratory/company (1% did not provide information on their practice). Laboratories testing smaller panels (<20 genes) were more inclined to use in-house commercial pipeline tools.

The majority of laboratories employ alignment tools based on the Burrows−Wheeler Alignment [17] algorithm, but other aligner strategies were also used (Supplementary Fig. 1a). For the variant identification the Genome Analysis Tool Kit [18] tools were most frequently used by the participants for variant calling (Supplementary Fig. 1b).

### Concordance analysis

**Fig. 4:**
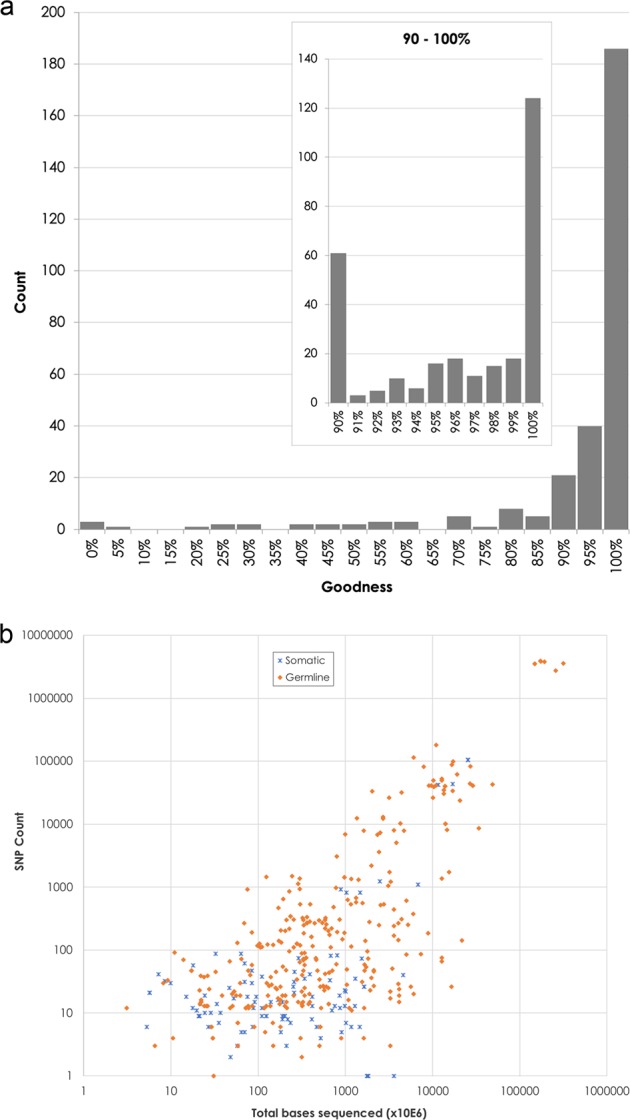
**a** Distribution of submissions from the 2016 germline scheme stratified by ‘goodness’ (correct calls/submitted calls, in %). **b** The number of identified SNPs as a function of bases read (from 2016 germline and somatic schemes)

The participant laboratory’s variant analysis data were compared against the relevant truth sets to determine the sensitivity of their analysis. In 2015 the majority of laboratories detected the same variants when the same genes were tested with 74% of laboratories getting >80% sensitivity for variant results calls (not shown). This was improved in 2016 with 89% of laboratories getting >80% sensitivity for variant results calls (Fig. 4a). F-scores > 0.80 for germline 2016/2017 was 83%/93% of laboratories, and for somatic 43%/71% respectively. F-scores > 0.95 for germline 65%/82% of laboratories, and for somatic 33%/43% in 2016/2017 respectively.

The effect of the tight selection criteria used to generate the consensus truth sets (at least seven observations, at least 75% agreement) was that genomic locations that had been assessed by less than seven laboratories, but still concordant among those laboratories, did not enter the truth set nor the assessable region. Such genomic locations were consequently only assessed against the EQA truth set (in 2016), while in 2017 they were assessed as FPs. Another consequence of the 75% consensus acceptance criteria is that some genomic locations with seven or more observations had a majority true variant agreement but were nevertheless only assessed against the EQA truth set (in 2016) or assessed as FP (in 2017) because of the 75% agreement not being reached. A limitation of this approach is that these genomic locations will likewise generate an FP classification for those who have found the true variants. As an example, in 2016 a number of laboratories were outliers in our analyses. Some laboratories demonstrated low concordance (Fig. 4a) particularly in the smaller panel size category (lower left section of Fig. 4a) due to either failing to identify the correct variants, or the reported ROI does not match the one declared by the participant in the BED file. The latter is demonstrated by some submissions reporting variants in a subset of their ROI. Since the concordance is calculated for the declared ROI as per the BED file, then the sensitivity score was detrimentally affected from a too wide declared ROI as compared to the ROI used by the laboratory.

Another observation based on the submitted data relates to the total amount of sequence generated for the variant calling process. The efficiency of the process can be calculated by considering the amount of raw data generated per declared variant (Fig. 4b). This will be different for the germline tests and for the somatic tests, and the expected allele frequency of the variants will have a bearing on the number of bases that are required for its identification. Despite these reservations, and the fact that this does not provide an estimation of the quality of the performed test, this nevertheless gives an indication about how much the costs can vary per called variant. As can be seen in Fig. 4b for the detection of 50 variants (ca 15 genes), there is an almost 1000-fold difference between the extreme cases in terms of total number of bases sequenced. It can also be seen that those laboratories that perform WES or WGS have efficiently optimised their assays since there is very little variation in number of bases sequenced per identified variant (upper right-hand corner of Fig. 4b). A laboratory should, on a regular basis, use robust validation procedures with known reference samples to find the optimal trade-off between cost and quality. Continuous quality control on informative quality measures can be used to provide an early alarm of drift in the quality and confidence of the reported results.

## Discussion

EQA is an essential part of the laboratory quality improvement process and an important component of laboratory accreditation. Traditionally EQA schemes in genetics and molecular pathology have focussed on targeted testing of single, or small panels of genes [19–21]. Development of an EQA activity for NGS has been very challenging due to the complexities of big data and large variation in the NGS approaches employed. For example, germline testing requires shallow sequencing depth, but with the breadth of a large target, e.g. larger gene panels, clinical exome, whole exome or whole genome. In contrast, somatic testing requires high read depth to detect variants present at low allelic frequencies in the samples but focuses on targeted hot spots providing information for clinical management. For germline testing addressing mosaicism, level of detection below the 50% heterozygous allele frequency may be needed, which improves with increased read depth. For somatic testing the clinical utility of detecting low allelic frequency pathogenic variants (often less than 1%) is unknown (except for liquid biopsies). At low frequency, the signal to noise ratio weakens and the risk for spurious artefactual detection increases. The data collated through the EQA described in this paper confirm these differences in approach and consequently the value of delivering two versions of this NGS EQA scheme which were tailored to the specific contexts of germline and somatic testing.

The design of the germline scheme is suitable for measuring the quality of NGS in a large and diverse group of participant laboratories. Theoretically, any DNA sample could be used if sufficient numbers of laboratories participate to ensure statistical power in the analysis. However, many well-defined reference samples are also available and could be used together with the participant consensus data. The power of these germline-focussed NGS EQAs enables the provision of unique EQA material for testing, and consequently generate a new reference genome sequence based on the analysed participant data. However, for somatic testing this was less straightforward. We explored several different material options using artificial reference materials but settled in 2017 on using material derived from a real patient tumour sample which contained approximately 200 variants thereby widening the scope for the participants. Future versions of the somatic scheme will need to ensure even wider clinical applicability by including more variants in different genes.

There is a need for reference materials that provide a highly accurate set of genotypes across a genome to help measure accurate variant calling. The US National Institute of Standards and Technology developed the ‘Genome in a Bottle’ standards [22] to help meet this need and Illumina developed its Platinum Genomes [23]. Whilst these enable the accurate measurement of sensitivity and specificity, they are becoming too familiar to laboratories and thus will no longer represent a blinded test. Furthermore, as these reference genomes are being used as the truth set for algorithm development, there is a significant risk that tools developed using this information become biased towards the reference the genomes they were trained/developed on i.e. assessing the results using the reference sequence used to develop the protocol. Using the methodology that we have developed in this EQA, we have demonstrated that it is possible to generate a new reference genotype from any sample, including those in publicly available biobanks. We acknowledge that >70% of laboratories which participated in our EQA used an Illumina sequencing platform and a large majority use GATK for variant calling, and therefore there may be an inherent bias in our process. However, we consider that this bias is a true reflection of current laboratory practice and will only change when new NGS technologies, reference genomes and testing algorithms become available. Consequently, our EQA may offer opportunities for participant laboratories to expose their bioinformatics pipelines to novel reference materials, thereby improving the quality of their testing process.

Accurate variant calling is dependent on efficiently capturing the targets, good quality sequencing and careful downstream bioinformatics. Published BPG [5, 6] have established a large number of different internal quality control (IQC) metrics which can help laboratories to understand the factors that affect the quality of NGS, as well as highlight any problems both with wet-laboratory sample processing and with dry-laboratory data processing steps. These guidelines include recommendations for validation or verification, as well as for systematic and regular quality control on all the bioinformatics files (FASTQ, BAM, VCF) for every diagnostic sample. During the processes of test optimisation and familiarisation, and validation, control quality metrics thresholds for all steps including the bioinformatics pipeline should be established so that they can be systematically applied to monitor the quality of all diagnostic samples [6]. These metrics, however, do not provide impartial external assessment. By incorporating a subset of these IQC metrics into the EQA scheme, a methodology which allows participating laboratories to benchmark the quality of their data against their own normal diagnostic samples as well as all other laboratories using the same platform and/or kit has been developed and provided. This analysis is helpful for understanding the full wet-laboratory and bioinformatics pipeline and its critical weaknesses in a given NGS strategy and will further facilitate improvements in NGS testing quality.

The baseline human genome reference sequence, maintained by the Genome Reference Consortium [24], is a consensus assembly from more than one individual used to provide basic genomic coordinates for each chromosome, but it does not represent a completed reference. This, in combination with the fact that many laboratories do not use a unique and stable sequence reference that is independent of updates to the reference genome assembly, such as the Locus Reference Genomic (LRG) [25, 26], means that there is more variability in reporting of disease-causing variants. This source of variability was managed by allowing the use of both GRCh37 and GRCh38 assemblies in 2017; 4% of the submissions were on GRCh38, half of them from WGS.

Another common source of variability encountered was differences in the interpretation of data standards [27]. The FASTQ file format is now well defined having resolved an early inconsistency with the encoding of quality scores. Whilst the bioinformatics community owes a great debt to the 1000 Genomes Project [28] that devised the SAM and VCF formats and developed samtools, bcftools, VCFtools and other software tools sets, the specifications for VCFs cover multiple needs and there might now be a case for a ‘clinical VCF’ building on the gVCF standard now supported by most tools. The VCF file format, version 4.2 [15] specifications are both liberal and complex and allow for a wide variety of different syntaxes for declaring the same information, creating hurdles for automated computational analysis, and resulting in different variant callers outputting quite different codes for the same variants. Multiple nucleotide variants and indels located very near to each other pose problems as they can be expressed in different ways [29]. Normalisation may not handle all edge cases, and this may lead to errors in the concordance analysis. We suggest that a stricter definition of how to convey information about genotype, about read depth and about allele frequency should be considered in future updates of the VCF format specifications. All three parameters are of cardinal importance in clinical reporting of variants.

During the assessment process we observed that some commercial software providers apply their own modifications to the output standards, most notably the removal of some important quality metrics from the BAM file by the JSI medical systems software [30] (e.g. mapping quality, off target % and error rate are considered valuable parameters by CAP and EuroGentest but absent from the JSI BAM files). Most other issues concerned the VCF files and a few issues stand out, particularly as they are of importance in diagnostic tests. For example, while remaining within the liberal boundaries of the VCF format definition, Qiagen (former CLC Bio) [31] applies a rare syntax for the genotype (GT). As described in the results, errors were also identified in some BED files.

Developing a standard for an EQA for NGS in clinical diagnostics presents a variety of problems. Firstly, due to the wide breadth of strategies used by laboratories, it was not feasible or useful to restrict the EQA to particular diagnostic testing scenarios since this would significantly limit the number of participating laboratories. As the aim of the EQA was to be a technical NGS assessment, then this was not a suitable approach. Therefore, we aimed for a truth set covering as many genes as possible. Secondly, different DNA extraction, capture/amplification, and library preparation methods, sequencing platforms and analysis pipelines all have inherent bias with the potential to produce variation in the observations, making the development of a truth set difficult. To minimise any bias, the EQA materials were validated using a variety of NGS platforms. In 2015 and 2016 this was done on behalf of the EQA providers, whilst in 2016 and 2017 the participant results were also used (stemming from multiple different NGS platforms). In 2016 the two differently derived truth sets (EQA-derived versus participant-derived) demonstrated good concordance with an F-score of 0.957. Such concordance, despite the very strict rules we applied, is made possible because of the large number of participants using different measurement technologies. When the truth set is derived from a participant consensus, a number of errors may be introduced. In particular, if there is a systematic error that leads to a large proportion of participants agreeing on an erroneous variant, it could be incorporated in to the truth set. In this case, some variants classified as FNs will not actually be FNs. Conversely, if there is a large proportion of participants that do not correctly call a variant, it may be left out from the truth set. In this case, some true variants will be classified as FPs. Further efforts will be needed to improve the approach to generate a participant consensus from such a large set of data as is generated by more than 400 participants.

Finally, this paper has evidenced that a broad range of software solutions are available to deliver specific parts of the analytical pipeline. The generation of the raw read data (FASTQ format) from the sequencer signal (primary analysis) is systematically handled by the sequencer software. The secondary analysis covers two critical steps: alignment of the reads to the reference genome and calling of the variants from the alignment. The final step (tertiary analysis) covers variant annotation and classification which can be performed manually for very small panels of genes but requires adapted variant interpretation software for larger gene panels. The observed lack of consensus in bioinformatics tools is understandable, but we consider it worrisome to observe that many pipelines were relying on tools which are considered to be outdated and/or no longer supported (e.g. Illumina MiSeq Reporter™ software v1.0—data from the 2017 schemes). This may introduce yet another layer of bias among the types of variants reported. We recommend that laboratories pay more attention to the version of the software they are using. We also recommend that laboratories ensure they introduce a stringent procedure for internal validation using internal or commercially available reference standards, regular verification and continuous quality control of the bioinformatics pipelines through the quality control of all samples [32] to be enacted between their EQA rounds.

In conclusion, we have established an EQA scheme for NGS workflows that is generic, technology and testing context agnostic, and which seamlessly integrates with laboratory testing pathways and bioinformatics analysis without introducing the need for new processes or files. This EQA is therefore suitable for germline as well as somatic variant testing settings covering the full range from single gene testing to full genome analysis. Theoretically, it could be extended to any discipline-specific EQA scheme (e.g., microbiology, virology, microbiome, etc.) where the primary focus is establishing interlaboratory comparison of the NGS testing process. The EQA results presented indicate that the laboratories participating in the 2015, 2016 and 2017 assessments are providing an ever-improving high-quality NGS service. However, there are still multiple challenges facing EQA delivery in this field; assessing large genomic rearrangements (structural variants, SV), copy number changes, (copy number variants, CNV), dealing with pseudogenes, GC rich and repetitive sequence regions, selecting performance criteria and applying quality thresholds, perhaps differentially over different parts of the genome, comparing similar NGS approaches between cohorts with meaningful numbers, sample sourcing and dealing with evolving and nonstandardised data formats. External pressure applied on the commercial manufacturers of the NGS systems and other interested parties is required to further harmonise and standardise these data types. This will result in better data quality, and facilitate improved data sharing among laboratories, which eventually will aid in delivering higher standards of NGS testing in patient clinical test pathways.

## Supplementary information

Figure legends

Supplementary Table 1: Capture kits used by participants

Sequence aligners used by participants (data combined for all three runs)

Variant callers used by participants (data combined for all three runs)
