# Supplementary material for: One byte at a time: evidencing the quality of clinical service next-generation sequencing for germline and somatic variants
Source: Eur J Hum Genet. 2019 Sep 30;28(2):202–12. doi: 10.1038/s41431-019-0515-1 (PMC6974611; doi:10.1038/s41431-019-0515-1)
Supplement: Supplementary file 1 — Figure legends [file 41431_2019_515_MOESM1_ESM.docx]

Supplementary Figure 1a: Sequence aligners used by participants (data combined for all three runs)

Supplementary Figure 1b: Variant callers used by participants (data combined for all three runs)

Supplementary Table 1: Capture kits used by participants
