# Supplementary figures and images for: One byte at a time: evidencing the quality of clinical service next-generation sequencing for germline and somatic variants
Source: Eur J Hum Genet. 2019 Sep 30;28(2):202–12. doi: 10.1038/s41431-019-0515-1 (PMC6974611; doi:10.1038/s41431-019-0515-1)

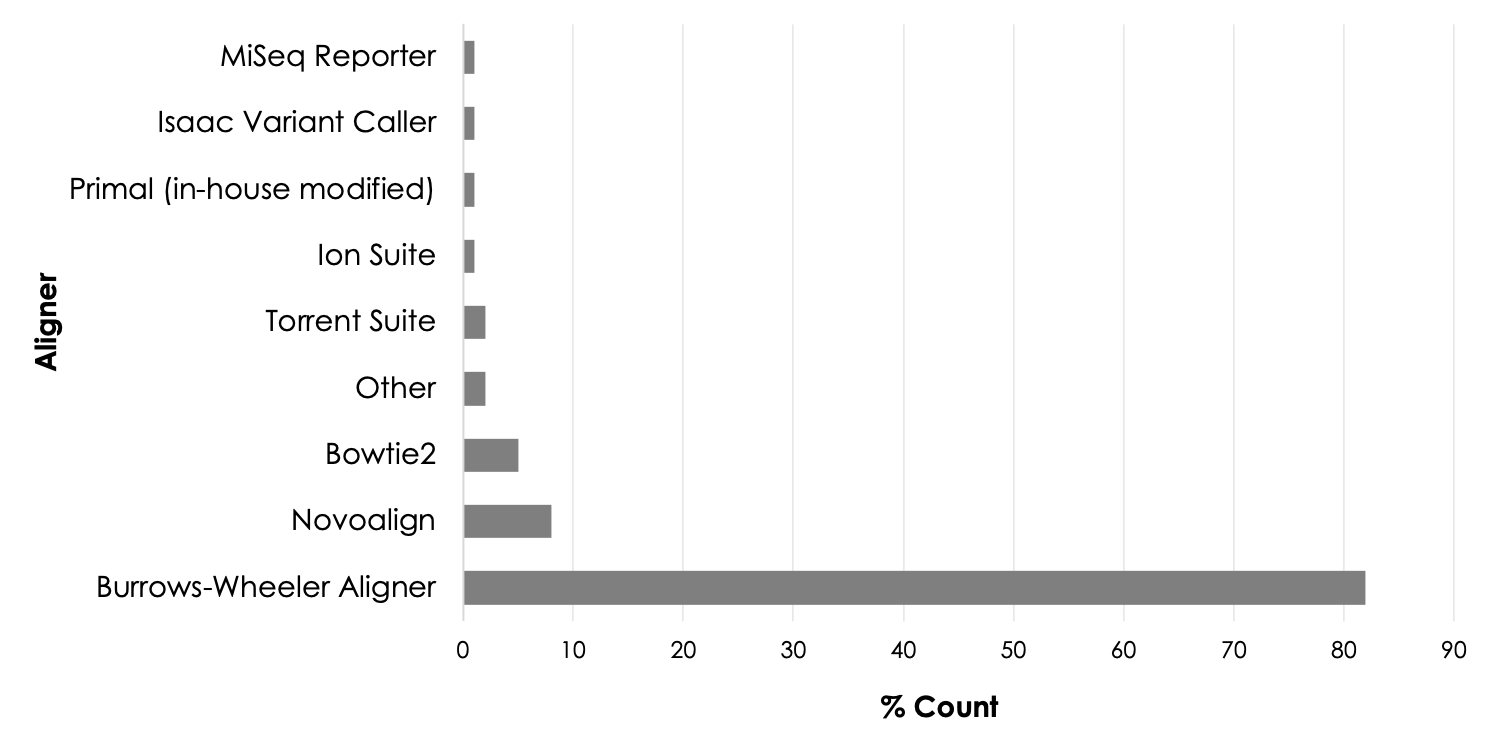

Supplement: Supplementary file 3 — Sequence aligners used by participants (data combined for all three runs) [file 41431_2019_515_MOESM3_ESM.png]

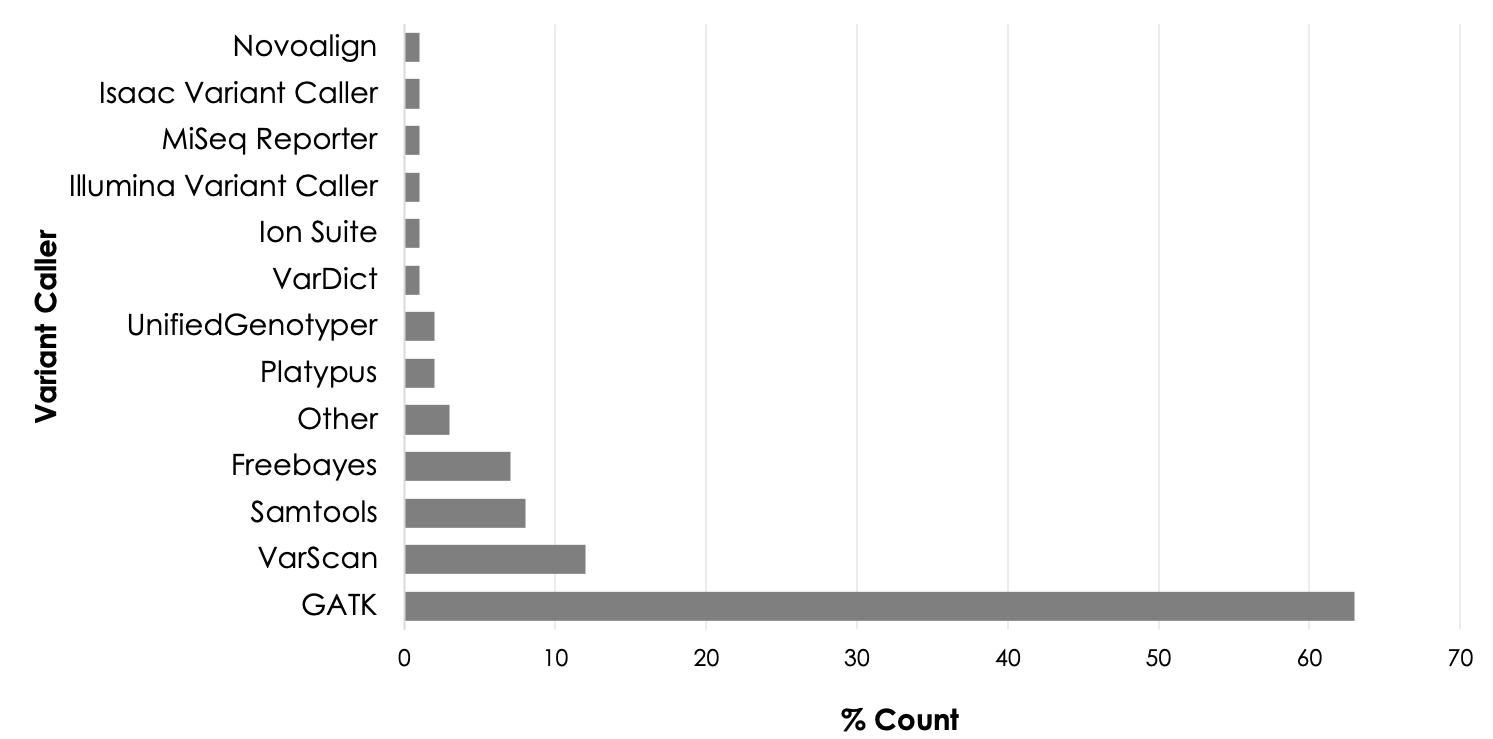

Supplement: Supplementary file 4 — Variant callers used by participants (data combined for all three runs) [file 41431_2019_515_MOESM4_ESM.png]
